# *Orf* virus (ORFV) infection in a three-dimensional human skin model: Characteristic cellular alterations and interference with keratinocyte differentiation

**DOI:** 10.1371/journal.pone.0210504

**Published:** 2019-01-30

**Authors:** Mahmod Muhsen, Martina Protschka, Laura E. Schneider, Uwe Müller, Gabriele Köhler, Thomas M. Magin, Mathias Büttner, Gottfried Alber, Sabine Siegemund

**Affiliations:** 1 Institute of Immunology/Molecular Pathogenesis, Center for Biotechnology and Biomedicine, College of Veterinary Medicine, University of Leipzig, Leipzig, Germany; 2 Institute of Pathology, Klinikum Fulda gAG, Fulda, Germany; 3 Institute of Biology, Division of Cell and Developmental Biology, University of Leipzig, Leipzig, Germany; NYU Langone Medical Center, UNITED STATES

## Abstract

ORF virus (ORFV) is the causative agent of contagious ecthyma, a pustular dermatitis of small ruminants and humans. Even though the development of lesions caused by ORFV was extensively studied in animals, only limited knowledge exists about the lesion development in human skin. The aim of the present study was to evaluate a three-dimensional (3D) organotypic culture (OTC) as a human skin model for ORFV infection considering lesion development, replication of the virus, viral gene transcription and modulation of differentiation of human keratinocytes by ORFV. ORFV infection of OTC was performed using the ORFV isolate B029 derived from a human patient. The OTC sections showed a similar structure of stratified epidermal keratinocytes as human foreskin and a similar expression profile of the differentiation markers keratin 1 (K1), K10, and loricrin. Upon ORFV infection, OTCs exhibited histological cytopathic changes including hyperkeratosis and ballooning degeneration of the keratinocytes. ORFV persisted for 10 days and was located in keratinocytes of the outer epidermal layers. ORFV-specific early, intermediate and late genes were transcribed, but limited viral spread and restricted cell infection were noticed. ORFV infection resulted in downregulation of K1, K10, and loricrin at the transcriptional level without affecting proliferation as shown by PCNA or Ki-67 expression. In conclusion, OTC provides a suitable model to study the interaction of virus with human keratinocytes in a similar structural setting as human skin and reveals that ORFV infection downregulates several differentiation markers in the epidermis of the human skin, a hitherto unknown feature of dermal ORFV infection in man.

## Introduction

Organotypic cultures (OTC) have been established for various purposes and can be a suitable equivalent, e.g. for studies of wound healing or infectious diseases manifesting in the skin [1–4]. Parapoxviruses (PPV) are epitheliotropic viruses infecting the skin and cause localized lesions. In contrast to systemic Orthopoxvirus infections, the human zoonotic infection with PPV, especially with Orf virus (ORFV), is a localized event resulting in a normally benign lesion commonly known as milker’s nodule that is completely resolved within a few weeks [5]. The take and manifestation of ORFV infection is dependent on a defective skin barrier or wounds to allow virus entry [5,6]. In some cases of human infection, severe immune suppression can lead to a highly vascularized, exceptionally proliferative weeping nodule, named giant Orf [7,8]. Histological examination shows edema of the dermis, capillary proliferation, acanthosis, necrosis and reticular degeneration of keratinocytes and intracellular eosinophilic inclusion bodies [9,10]. *In vitro* infection with ORFV is usually performed in classical cell cultures using primary cells from sheep or ruminants for virus isolation and propagation. Regarding skin-derived primary keratinocytes or dermal fibroblasts, limited studies were undertaken with ORFV to evaluate cytopathic effects or transcriptomic profiles [11,12]. The infection of OTC generated from ovine foreskin primary lamb keratinocytes with ORFV resulted in ballooning degeneration of cells in the superficial layers [1]. In the natural host ORFV uses a number of virus-specific escape proteins, including interferon resistance factor, chemokine binding protein (CBP), IL-2, granulocyte/macrophage growth inhibitory factor (GIF) and viral IL-10 [13–19] to counteract the immune response. An exceptional and unique feature of ORFV is the expression of vascular endothelial growth factor E (VEGF-E) promoting angiogenesis [20,21]. In a mouse model virus-encoded VEGF-E induces dermal vascularization and significantly increases the number of keratinocytes within the skin [15].

Since the majority of reports about zoonotic Orf infection in man deal with histological examination of established lesions, we were interested in zoonotic ORFV infection and virus-linked effects on differentiating vs. already differentiated epidermal cells present in OTC generated from primary human foreskin keratinocytes. *In vitro* infection of OTC allows the follow-up of virus-induced alterations in well-defined cell populations of the epidermis without the influence of the immune system. For analysis of ORFV-induced effects on keratinocyte differentiation, the expression of the differentiation markers keratin 1 (K1) and keratin 10 (K10), the transcription of the late differentiation marker loricrin, as well as presence of keratin 14 (K14) found in proliferating basal keratinocytes [22–26], were determined after ORFV infection of OTC and human foreskin explants. Moreover, ORFV-induced effects on proliferation were investigated.

## Materials and methods

### Virus

ORFV B029 strain was isolated from an infected human skin biopsy collected after transmission from sheep in a herd suffering from an Orf outbreak in 1996 in Germany [27,28]. Virus purification was done by sucrose gradient centrifugation [29].

### Cell culture

Primary normal human keratinocytes (KC) were isolated from epidermis of juvenile foreskin as described by Saalbach *et al*. [30]. Briefly, the foreskin was incubated in dispase (2.2 U/mL Dispase II; Roche, Mannheim, Germany) overnight at 4°C for epidermis separation. Single cells were then isolated by 0.05%Trypsin/0.02% EDTA digestion (Biochrom, Berlin, Germany) for 9 minutes at 37°C, followed by vortexing, filtration with a 40 μm cell strainer and cultivation in KGM-Gold KC medium (Lonza, Cologne, Germany). The study was approved by the ethics committee of the University Leipzig (269/16-ek).

3T3-J2 cells, immortalized mouse fibroblasts, were used as feeder cells for KC in the 3D culture [31] and grown in Dulbecco’s Modified Eagle Medium (DMEM) (Biochrom) supplemented with 10% fetal bovine serum (FBS) (Gibco, Thermo Fisher Scientific, Schwerte, Germany). Calf oesophagus cells (KOP cells, RIE 244, obtained from Friedrich-Loffler-Institute, Isle of Riems, Germany), which are highly permissive for ORFV, were used for detection of viable virus from OTC and foreskin. KOP cells were grown in MEM Hanks medium (Merck, Darmstadt, Germany) supplemented with 5% FBS (Thermo Fisher Scientific), 1% non-essential amino acids (NEA), 1% sodium pyruvate (100 mM), 0.7% sodium bicarbonate (7.5% solution) and 1% glutamine (all from Merck). The cells were incubated at 37°C in a humidified cell culture incubator in the presence of 5% CO_2_.

### Preparation of the 3D organotypic culture (OTC)

The collagen gel was prepared by mixing 3T3-J2 cells with collagen type I (Corning Inc., Corning, NY; USA), reconstitution buffer (1 g NaHCO_3_ and 2.3 g HEPES in 50 mL 0.05 N sodium hydroxide) and 10 x DMEM (Biochrom). The 3T3-J2-collagen mixture was distributed in 12-well culture plates. After solidification, the cells were grown submerged with DMEM for four days. The KC were seeded onto the 3T3-J2-collagen gel and covered with E-medium supplemented with epidermal growth factor (EGF). The E-medium consisted of 458.5 ml DMEM (Biochrom) and 458.5 ml DMEM/nutrient mixture F-12 Ham (Sigma Aldrich, Munich, Germany), supplemented with 10 ml E cocktail mixture (10 μM adenine, 5 μg/ml bovine pancreatic insulin, 5 μg/ml human apo-transferrin and 5 μg/ml triiodothyronine), 10 μg/ml gentamicin, 0.25 μg/ml amphotericin B), 0.4 μg/ml hydrocortisone, 10 ng/ml cholera toxin (all from Sigma Aldrich) and 5% FBS per liter. The cell number seeded was 4x10^5^ 3T3-J2 cells and 5x10^5^ KC per well of a 12-well culture plate [4]. After incubation for 2 days, the culture gels were raised onto sterilized raft metal grids, placed in 60 mm petri dishes, and an air-liquid interface was formed by adding the E-medium without EGF to the bottom of the dish touching the collagen gel at the bottom. The OTCs were incubated for 10 days at 37°C with a change of E-medium every other day and then frozen for analysis (S1 Fig).

### Protocol of virus infection

OTCs were infected with ORFV B029 at day 0 before KC start to differentiate just before lifting the culture on rafts to the air-liquid interface (d0-10), or at day 8, i.e. 2 days before termination of the OTC setup (S2 Fig).

The infection was performed by applying the virus onto the top cell layer. For (d8-10) experiments, OTC were wounded by incision prior to application of the virus suspension. Infected OTC were then maintained until day 10. The virus multiplicity of infection (MOI) was calculated according to the number of KC forming a complete monolayer at the day 0 before lifting on the raft. OTC were inoculated with ORFV at MOI~3 and incubated for two hours for absorption. The OTCs were then washed twice with E-medium and maintained at the air-liquid interface condition.

For foreskin infection, an area of about 1 cm^2^ of foreskin epidermis was carefully incised in depth of max 1 mm using a sterile scalpel prior to infection. The virus solution (3x10^6^ particles) was then applied onto this area and pre-incubated for 2 hours. After this 2-hour adherence period, the infected foreskin explant piece was washed twice with phosphate buffered saline (PBS) to remove extracellular excess virus particles and then further incubated at 37°C for another 48 hours until analysis. OTC and foreskin were washed twice with PBS before freezing.

Infection of a 2D monolayer KC culture was done in 6-well plates (for flow cytometry analysis) or 24- respectively 12-well plates (for RT-qPCR analysis) at 90–95% confluence in KGM Gold Medium. ORFV was added for 2 h at MOI~3 to KC and non-attached viral particles were removed by washing twice. The KC were then incubated for 48 h.

### Virus re-isolation

Detection of viable ORFV after infection was performed by co-cultivation of supernatant from homogenized OTC tissue or foreskin cryosections with KOP cells, a highly permissive cell for ORFV infection, in 12-wells-plates. For homogenization 30 slices of frozen OTC or foreskin sections (4 μm thin) of 20 mm^2^ were collected in 500 μl PBS and 3 times freeze-thaw treated. The debris of the OTC or foreskin sections and the OTC homogenate was removed by centrifugation (1,000 rpm, 10´). Supernatants were diluted 1:10 and co-cultured with KOP cells. The KOP monolayers were analyzed for cytopathic effects (CPE) under a light microscope (ZEISS Axiovert 40c). When a CPE became visible, for selected samples the presence of ORFV was confirmed by staining for ORFV envelope protein using an ORFV-specific mouse monoclonal antibody.

### Histological analysis of OTC and foreskin

The OTCs and foreskin were embedded in tissue freezing medium (Jung, Nussloch, Germany), and snap-frozen with 2-methylbutan (Roth, Karlsruhe, Germany) on dry ice before preserving at -80°C. Cryostat sections of 4 μm thickness were mounted on glass slides, dried for 2 hours at room temperature (RT), stained with hematoxylin (Roth, Karlsruhe, Germany) and eosin (Sigma Aldrich) (H&E), and monitored using an AX70 microscope (Olympus, Hamburg, Germany).

### Immunofluorescence staining

Cryosections of OTC and human foreskin sections were fixed with Roti-Histofix and permeabilized with 0.3% Triton X-100 (both from Roth, Karlsruhe, Germany) in PBS. Blocking was performed with 10% normal donkey serum (Dianova, Hamburg, Germany) in PBS with 0.025% Tween 20 (PBST) for one hour at RT. The sections were incubated with primary antibodies at 4°C overnight and with secondary antibodies for 1 hour at RT for immunofluorescence staining (Table 1). The K10-specific antibody (working dilution 1:200) was used for OTC and foreskin sections in the single stain experiment, whereas K1-specific (working dilution 1:800) and keratin 14 (K14)-specific antibodies (working dilution 1:200) were used for single and double staining.

**Table 1:** Antibodies for immunohistochemistry and flow cytometry.

primary antibodies	company	secondary antibodies	company
**anti-K1** polyclonal rabbit IgG	PSL (Heidelberg), [32]	Cy3-conjugated donkey IgG anti-rabbit IgG	Dianova #711-165-152
		Poly-HRP-conjugated goat anti-rabbit antibody*	Thermo Fisher #B40943
		Alexa Fluor 488-conjugated donkey IgG anti-rabbit IgG	Dianova #711-545-152
**anti-K10** monoclonal mouse IgG1	PSL (Heidelberg), [32]	Cy3-conjugated goat IgG anti-mouse IgG	Dianova #115-165-146
**anti-K14** polyclonal rabbit IgG	PSL (Heidelberg), [23]	Cy3-conjugated donkey IgG anti-rabbit IgG	Dianova #711-165-152
**anti-ORFV (6E8)** monoclonal mouse IgG2a	Santa Cruz Biotechnology # SC-101590X	Cy2-conjugated donkey IgG anti-mouse IgG	Dianova #715-225-150
**anti-loricrin** polyclonal rabbit IgG**	BioLegend 905104	Poly-HRP-conjugated goat anti-rabbit antibody*	Thermo Fisher #B40943

*used for double staining

** used for staining shown in S3 Fig.

Double staining was done as described by Tóth and Mezey [33]. Briefly, after overnight incubation with the K1- or loricrin-specific antibody, a poly-HRP-conjugated goat anti-rabbit secondary antibody from the Alexa Fluor 488 Tyramide SuperBoost Kit (Thermo Fisher Scientific) was applied for 1 hour at RT. After removing excess antibody, the sections were incubated with Alexa Fluor 488 tyramide reagent for 5 minutes. This antibody-fluorescence complex was removed by hot 10 mM citric acid buffer. After washing, the K14-specific antibody was applied for 1 hour at RT and detected with a Cy3-conjugated donkey anti-rabbit IgG as described before. Nuclei were counterstained with Hoechst 33342 (Molecular probes, Eugene, USA). The slides were analyzed with a fluorescence microscope (Olympus IX81, Olympus) and the CellSens Dimension software (Olympus).

### RNA isolation and RT-qPCR analysis

In addition to transcription of *K1*, *K10*, *K14* and *loricrin*, the transcription of ORFV-specific genes was examined by reverse transcription-quantitative polymerase chain reaction (RT-qPCR). The early viral genes *IL-10-like protein* and *VEGF-like protein* [34], coding for VEGF-E; the intermediate gene transcription factor (*VITF-3*) [35] and the late expressed gene *B2L*, coding for the major envelope protein [36], were analyzed. The *Beta-2-microglobulin (B2M)* was used as reference gene. Briefly, the OTC were quick-frozen in liquid nitrogen and stored at -80°C until RNA extraction. Approximately 0.25 cm^2^ OTC tissue was ground in a liquid nitrogen-cooled mortar with pestle or homogenized with a Precellys 24 tissue homogenizer (VWR Peqlab; Darmstadt; Germany). Total RNA was isolated using 1 ml peqGOLD TriFast (VWR Peqlab). The concentration of the RNA was determined spectrophotometrically (NanoDrop, VWR Peqlab). The DNA was digested with one unit DNase/μg RNA (Thermo Fisher Scientific) for 30 min at 37°C. RNA was precipitated with 1/10 volume of 3 M sodium acetate, pH 5.5 and two volumes of 100% ethanol. Reverse transcription was performed using ‘High Capacity cDNA Reverse Transcription Kit’ (Applied Biosystems, Foster City, USA). Real-time PCR with SYBR green detection was performed using iQ SYBR Green Supermix (Bio-Rad, Munich, Germany) and an iQ5 multicolor real time PCR detection system (Bio-Rad). The relative quantification of the transcripts was done by the 2^(-ΔΔCt)^ method.

For the analysis of 2D monolayer KC culture cycle threshold (Ct) values were determined using cDNA corresponding to 0.5 ng (K14, PCNA), 5 ng (K1, K10, Ki-67) or 50 ng (loricrin) reversed transcribed RNA. The primers used for RT-qPCR analysis are shown in Table 2.

**Table 2:** Primers used for RT-qPCR analysis.

Name	Sequence	GenBank Accession No.	Position	Exon
HS_B2M_for	5´-TAAGTGGGATCGAGACATGTAAGC-3´	NM_004048.2	399–422	2/3
HS_B2M_rev	5´-GGAGCAACCTGCTCAGATACA-3´	NM_004048.2	627–607	4
ORFV_VEGF-like_for	5´-gaagttgctcgtcggcata-3´	KF837136.1	133541–133559	n.a.*
ORFV_VEGF-like_rev	5´-AACCGCTGAGAAGTCAGCTC-3´	KF837136.1	133708–133689	n.a.
ORFV_IL-10-like_for	5´-ACTTGGAAGAGGTGATGCCG-3´	AY231116.1	326–345	n.a.
ORFV_IL-10-like_rev	5´-TGCAGCATGTTGAACACACG-3´	AY231116.1	506–487	n.a.
ORFV_envelope_for	5´-AGTCCGAGAAGAATACGCCG-3´	NC_005336.1	11375–11394	n.a.
ORFV_envelope_rev	5´-GGGACCTCATGAACCGCTAC-3´	NC_005336.1	11521–11502	n.a.
ORFV_VITF-3_for	5´-CCACGACAGCTACCACACTT3´	NC_005336.1	88946–88965	n.a.
ORFV_VITF-3_rev	5´-CGGTACGTGATCTCGGTGAG-3´	NC_005336.1	89089–89070	n.a.
HS_K1_for	5´-TGGTGGAGGATTACCGGAAC-3´	NM_006121.3	844–863	2
HS_K1_rev	5´-CTGAGACAACTCTGCTTGGT-3´	NM_006121.3	1034–1015	4/5
HS_K10_for	5´-ATGGCAACTCACATCAGGGG-3´	NM_000421.3	580–599	1
HS_K10_rev	5´-ACCTCATTCTCATACTTCAGCCT-3´	NM_000421.3	751–729	2/3
HS_K14_for	5´-ACGCCCACCTCTCCTCC-3´	NM_000526.4	1329–1345	6/7
HS_K14_rev	5´-TTCTTGGTGCGAAGGACCTG-3´	NM_000526.4	1476–1457	8
HS_loricrin_for	5´-TTGGAGGTGTTTTCCAGGGG-3´	NM_000427.2	1024–1043	2
HS_loricrin_rev	5´-ACTGGGGTTGGGAGGTAGTT-3´	NM_000427.2	1196–1177	2
HS Ki-67 for	5´-GTCAAGAGGTGTGCAGAAAATCC-3´	NM_001145966	8791–8813	13
HS Ki-67 rev	5´-GCCGATTCAGACCCAGCAAA-3´	NM_001145966	9037–9018	14
HS PCNA for	5´-GATGTACCCCTTGTTGTAGAG-3´	NM_002592.2	933- 953	6/7
HS PCNA rev	5´-TGCTGGCATCTTAGAAGCAGTT-3´	NM_002592.2	1085–1064	7

* not applicable (n.a.)

### Flow cytometry

Human KC were harvested and stained with fixable viability dye eFluor 780 (eBioscience; now Thermo Fisher Scientific, Carlsbad, USA) according to the manufacturer instructions. After fixation in 2% paraformaldehyde, permeabilization was achieved using 0.5% saponin in PBS with 3% FBS and 0.1% sodium azide. The K1-specific antibody was used in combination with Alexa Fluor 488-conjugated donkey anti-rabbit IgG (Dianova). All incubation steps were performed for 30 minutes at RT. The cells were acquired on a BD LSRFortessa II (BD Bioscience, Heidelberg, Germany) and analyzed using FlowJo software (Flow Jo LLC, Ashland, Oregon)

### Statistical analyses

Statistical analyses were performed with the Graph Pad Prism 7 software (GraphPad Software Inc., San Diego, USA). The Shapiro-Wilk test was used to test for normal distribution. In case of passing the normality test, the 2 h values and 2 d values for transcription of ORFV genes were tested against each other using the one sample t-test. In case of K1 analysis by flow cytometry a one sample t-test was used to compare medium control against ORFV-infected samples.

## Results

### Epithelial structures of OTC and foreskin

**Fig 1:**
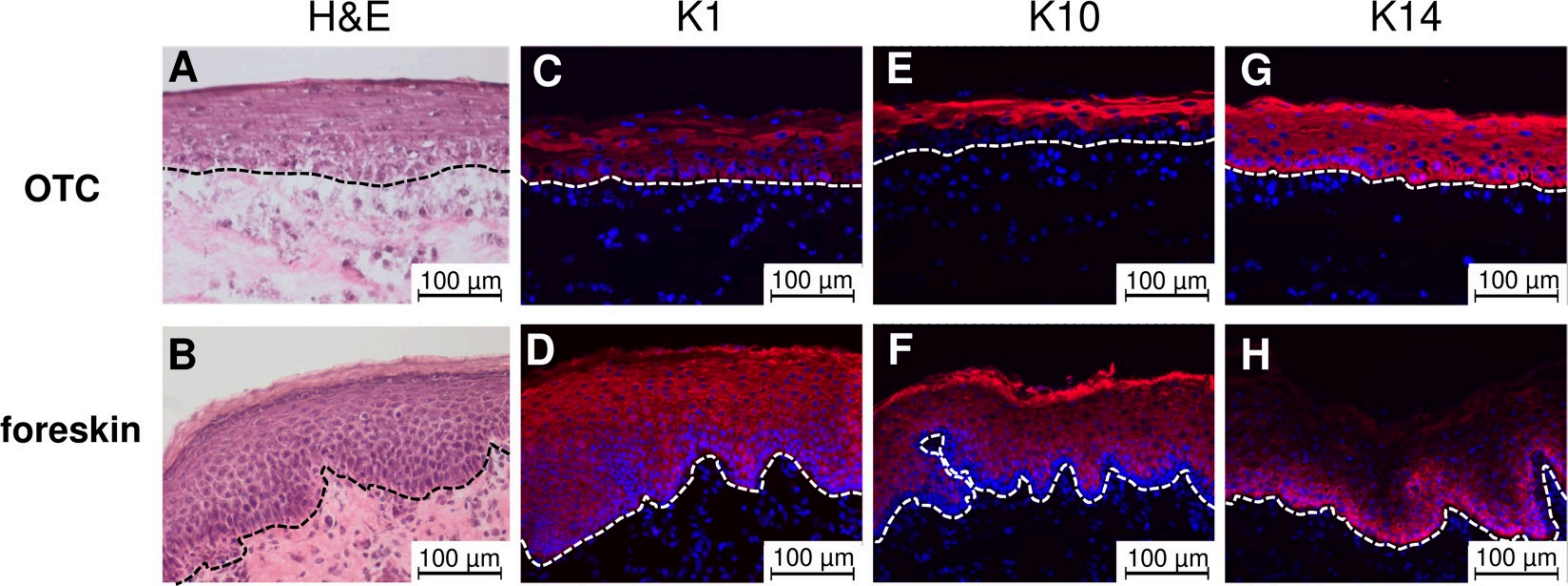
Analysis of skin structure and keratin expression. The epithelium of OTC consist of three distinguishable KC layers: polygonal shaped cells on top of the basal lamina followed by flattened KC in spinous and granular layers, followed by flattened corneocytes (A). Co-localized K1/K10 are located in the suprabasal layer (C and E). K14 is distributed all over the epidermis (G). Foreskin histology shows all four epidermal layers of human skin: *stratum basale*, *stratum spinosum*, *stratum granulosum* and *stratum corneum* (B). K1/K10 staining is located similar to OTC in the suprabasal layer with a stronger K10 stain in *stratum corneum* (D and F). K14 is strictly located in the cells of the *stratum basale (H)*. Keratins are displayed in red (Cy3) and nuclei are stained blue (Hoechst 33342). Representative H&E and immunofluorescence from three independent experiments (n = 3).

In order to investigate the effects of ORFV infection in human skin, we established a 3-dimensional (3D) organotypic culture (OTC) as skin model. OTC was developed from KC derived from human foreskin explants as described in methods (S1 Fig). After 10 days of culture at the air-liquid interface, the OTC displayed an epidermal morphology resembling human skin (Fig 1). The human skin consists of different layers of epithelial cells, i.e. the *stratum basale*, *stratum spinosum*, *stratum granulosum*, and *stratum corneum* [37]. In the OTC, a compact basal layer of keratinocytes at the bottom was overlaid by flattened keratinocytes. Flattened cells with visible granules formed the granular layer beneath keratinocytes located in the top layer (Fig 1A). Histology of the foreskin explants completely shows the physiological skin structure of hairless skin, including dermal papillae (Fig 1B). During their development from the *stratum basale* to the *stratum corneum*, keratinocytes undergo a differentiation process and change their expression profile of different keratins [38]. To analyze differentiation markers of KC in the OTC model, we compared the presence of K1 and K10, keratins produced during differentiation in suprabasal layers, and of K14, a keratin usually found in proliferating basal KC [25,38]. We observed a strong expression of K1 in the suprabasal layers of the OTC, whereas K1 expression in the *stratum corneum* was marginal (Fig 1C). Also in human foreskin explants, strong K1 expression was present in the suprabasal layers (Fig 1D). In contrast to OTC, K1 expression was also found in the *stratum corneum* (Fig 1C and 1D). K10 was predominantly expressed in the outer stratified layers of the OTC epidermis whereas expression in the basal keratinocyte layer was marginal (Fig 1E). Also in human foreskin biopsies, the K10 signal was strong in the outer stratified layers and weaker at the layers beneath (Fig 1F). Overall, the expression domains of K1 and K10 in suprabasal cells were overlapping, as expected.

Presence of K14, a marker for undifferentiated keratinocytes in the basal lamina [39], was visible throughout the whole thickness of stratified epidermis in the OTC (Fig 1G), whereas it was located in the layer of *stratum basale* in the foreskin sections (Fig 1H). Cell proliferation mostly takes place in basal cells and *in vivo*, proliferating cells are restricted to the basal layer with few exceptions. In the 3D model, there is an artificial delay in differentiation that may lead to more proliferation in suprabasal cells.

Despite the overall presence of K14 in OTC (S3C Fig), loricrin was present in the upper cell layers in OTC (S3A Fig), resembling loricrin expression of foreskin explants (S3B Fig). Taking together, our data show that the OTC consists of three distinguishable layers of keratinocytes with basal large polymorph shaped cells and a suprabasal layer of flat KC topped by keratinized cells and an expression profile of KC differentiation markers largely similar to human foreskin explants.

### ORFV-induced cytopathic effects

**Fig 2:**
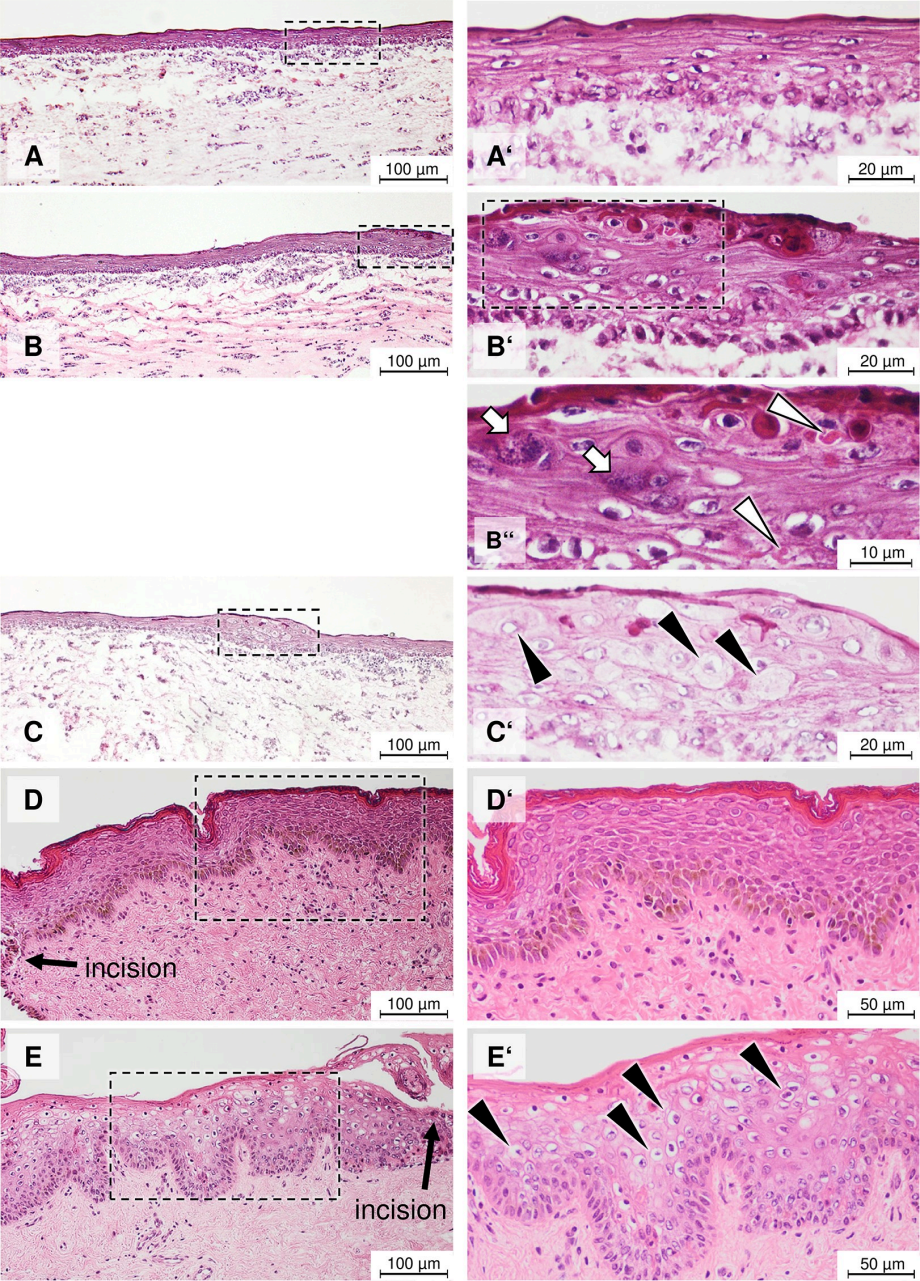
Restricted sites of ORFV-induced CPE in OTC and foreskin. H&E staining of epithelial structure of non-infected OTC (A and A’). ORFV infection (d0-10) causes thickening of epidermis and disorganization of the suprabasal layer at distinct sites of OTC (B) consisting of vacuolated KC (B’), ballooning degeneration of KC, and cytoplasmic eosinophilic inclusion bodies (B”). After a 2-day infection period (d8-10), a similar CPE is caused in OTC (C and C’). A well stratified epithelium with clear shape of KC is observed in non-infected foreskin (D and D’). Similar to OTC, vacuolization and ballooning degeneration of KC is present in the suprabasal layer (E and E’). H&E staining of 4 μm cryosections from three independent experiments (n = 3). Granule clumps in KC marked by open arrows. Eosinophilic inclusion bodies in B” marked by open arrow heads. Enlarged vacuolated KC in C’ and ballooning degeneration in E’ are marked by black arrow heads.

To study the impact of ORFV at the start of OTC development (i.e. during keratinocyte differentiation), the infection was performed at day 0. To follow the infection near the termination of OTC (i.e. near termination of keratinocyte differentiation), ORFV was applied at day 8 (S2 Fig). For comparison, uninfected OTC were analyzed (Fig 2A and 2A’). Even when infected on day 0, a marked CPE was visible at the termination of OTC at day 10 (Fig 2B’). The CPE is characterized by distinct local alterations such as a thickening of the *stratum corneum* known as hyperkeratosis, degeneration of keratinocytes, enlarged and spherical shape of keratinocytes (Fig 2B and 2B’) with increased granule clumps at the level of *stratum granulosum* (Fig 2”B, open arrows), and multifocal, cytoplasmic, eosinophilic inclusion bodies in the keratinocytes (Fig 2B”, open arrow heads). Also in OTC infected with ORFV at day 8, we observed local alterations such as increased thickness of the epidermis known as hyperplasia, ballooning and reticular degeneration in suprabasal epidermal layers (Fig 2C and 2C’, black arrow heads). Thus, our data indicate that ORFV induces CPE in OTC that is visible during differentiation of the keratinocytes (d0-10) as well as after a 2-day incubation period done with differentiated keratinocytes (d8-10).

To mimic the *in vivo* ORFV infection in wounded human skin, foreskin explant pieces were infected after a slight incision with a scalpel to expose damaged epidermal cells to the virus followed by an incubation period of 2 days. As control, incised foreskin explants were incubated for two days in medium. Non-infected skin explants displayed a normal stratified epidermal structure with clear-shaped keratinocytes and nuclei (Fig 2D and 2D’), whereas in infected foreskin sections, hyperplasia, ballooning degeneration of keratinocytes and shrinking of nuclei in suprabasal layers were apparent (Fig 2E and 2E’, black arrow heads). These observations resemble the ORFV-induced effects observed in the suprabasal layers of the OTC infected at day 0 and incubated until day 10 (i.e. during differentiation) as well as infected at day 8 and incubated until day 10 (i.e. near termination of differentiation). Thus, our data confirm the similarity of OTC and foreskin tissue in ORFV susceptibility and CPE formation. Moreover, the OTC data demonstrate that ORFV induced cellular alterations that appear to be independent of keratinocyte differentiation state.

### Detection of ORFV envelope protein and ORFV re-isolation

**Fig 3:**
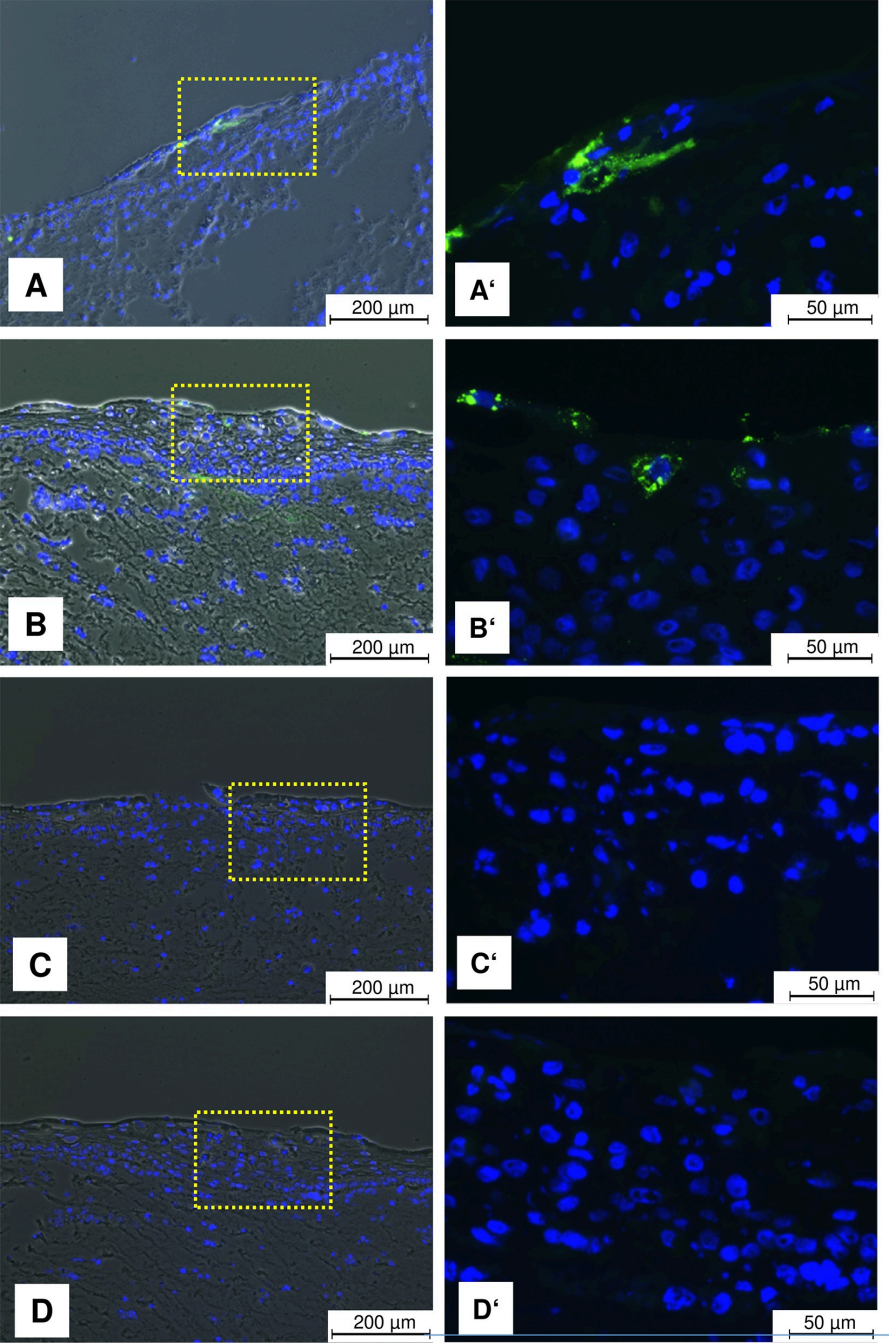
ORFV envelope detection in ORFV-infected KC. Representative fluorescence images show the localization of ORFV virus envelope protein (green) in few CPE-affected KC in suprabasal layer of OTC upon infection at day 0 (d0-10) (A and A’). Infection on day 8 (d8-10) produced more ORFV envelope positive cells (B and B’). Negative controls of nonspecific reaction by second antibody staining of OTC infected (d0-10) and (d8-10) are shown in images (C and C’) and (D and D’), respectively. The nuclei were stained with Hoechst 33342 (blue). A phase contrast image is overlaid for orientation (A-D).

To study the localization and presence of ORFV in OTC, OTC sections were stained with an ORFV envelope-specific antibody and analyzed by immunofluorescence. In OTC infected at day 0 (d0-10), the presence of virus envelope protein was restricted to the cytoplasm of a few damaged keratinocytes in locally affected epithelium in the upper epidermal layers, whereas no virus envelope signal was observed at distant sites without CPE alterations (Fig 3A and 3A’). In OTC infected at day 8 (d8-10), more infected KC were visible in the *stratum corneum* (Fig 3B and 3B’). This result indicates that ORFV is mainly present and maintained in the keratinocytes of the outer layers, does not spread laterally among neighboring KC, and can persist at least for 10 days during the generation of OTC. Although in infected foreskin a localized CPE was induced by ORFV, no virus-specific envelope protein was detectable as described for scarified ovine skin 48 hours after ORFV infection [40].

To test for replication competent ORFV still present in OTC and foreskin at the end of the infection period, tissue homogenates were co-cultivated with permissive KOP cells. Replication-competent ORFV was present in all tested samples of (d0-10)-infected OTC, (d8-10)-infected OTC, and foreskin explants infected for 2 days (Table 3). To demonstrate that the CPE in KOP cells was caused by ORFV, ORFV identity was confirmed by immunofluorescence staining of ORFV envelope protein (S4 Fig). Non-infected OTC and non-infected foreskin biopsies did not induce CPE in KOP cells.

**Table 3:** CPE caused by ORFV re-isolated from OTC and foreskin tissue.

	duration of infection (d)	Number of infected samples causing CPE in KOP / number of conducted experiments
**OTC**	2	3/3
	10	2/2
**foreskin**	2	4/4

Re-isolation of replication-competent ORFV after infection was done by co-cultivation of homogenized OTC tissue or foreskin with KOP cells. Shown are the duration of infection (first column), and the number of samples, from which replication-competent ORFV could be re-isolated in relation to the number of experiments performed.

### Transcription of ORFV genes in OTC

**Fig 4:**
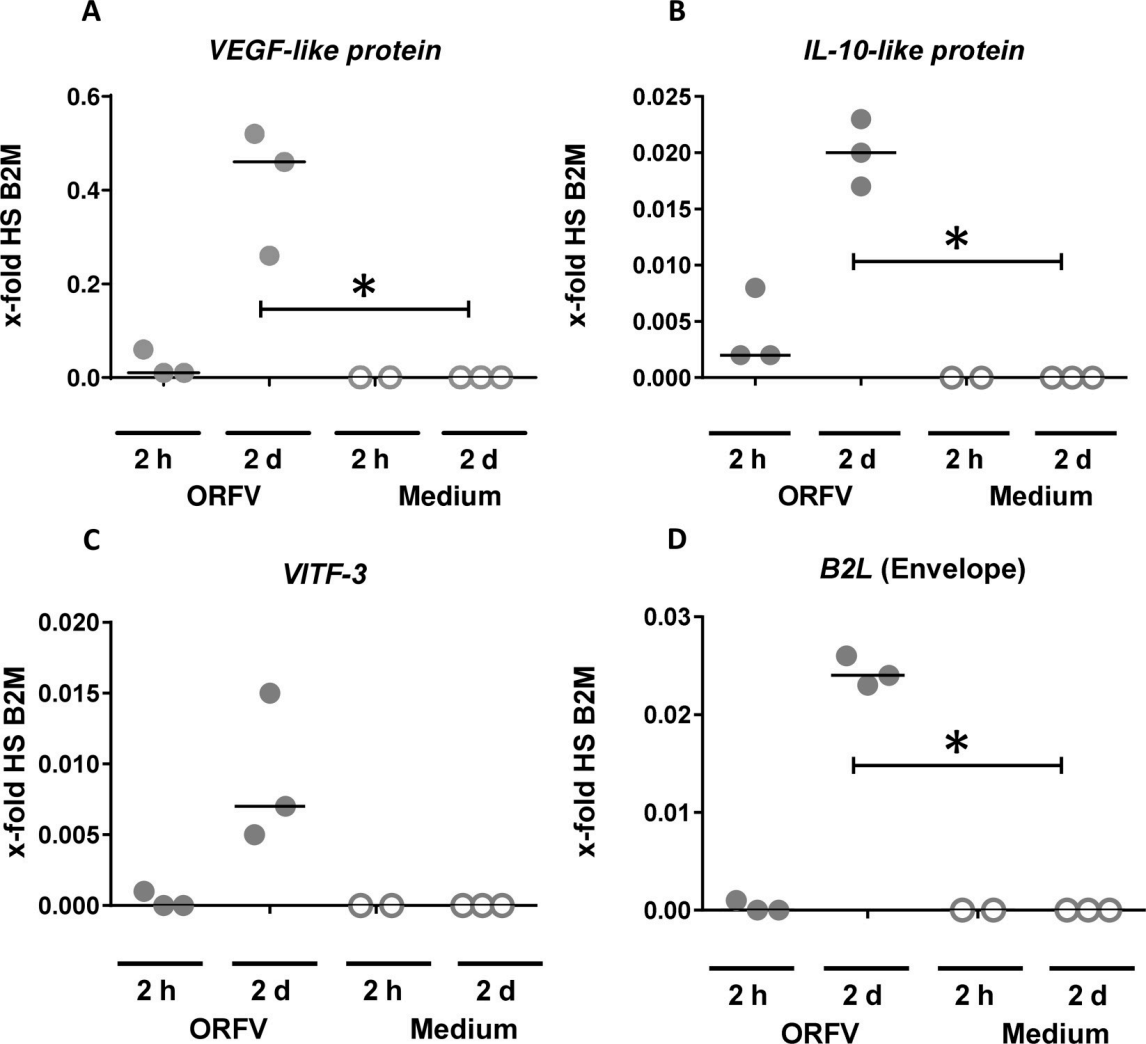
Transcription of ORFV-specific *VEGF-like protein*, *IL-10-like protein*, *VITF-3* and *B2L* in OTC. Relative quantification of early (A and B), intermediate (C) and late (D) genes of ORFV by RT-qPCR in OTCs (each dot represents a separate experiment). Induction of *VEGF-like protein*, *IL-10-like protein*, *VITF-3* and *B2L* (coding for major envelope protein) in OTCs following ORFV infection was measured by RT-qPCR. OTCs were infected at day 8 and analyzed 2 h and 48 h post infection. Values on the y-axes are calculated relative to human reference gene *beta-2-microglobulin* (*B2M*). For statistical analysis the normal distribution was calculated with the Shapiro-Wilk test. The ORFV 2 d data were normally distributed, they were tested for statistical significance in comparison to the reference (data: medium 2 d) with the one sample t test. *, p<0.05.

In addition to virus protein detection in infected OTC, we analyzed the transcription of viral genes to test the expression of early, intermediate and late viral genes. Therefore, we examined transcription of the ORFV-specific early genes *VEGF-like protein* and *IL-10-like protein*, the intermediate gene *VITF-3*, and the late expressed *B2L* (coding for major envelope protein) by RT-qPCR in infected and non-infected OTC. We observed a slight increase of transcription for the early genes *VEGF-like protein and IL-10-like protein* already 2 hours after OTC infection that became more pronounced after an incubation period of 2 days (d8-10) (Fig 4A and 4B). Until 48 hours after infection, the transcription of all four tested genes, including the late transcribed gene coding for ORFV envelope, increased (Fig 4). This indicates that at least one complete viral replication cycle takes place. No transcription was detectable in non-infected tissue.

### Reduced differentiation of keratinocytes upon ORFV infection

**Fig 5:**
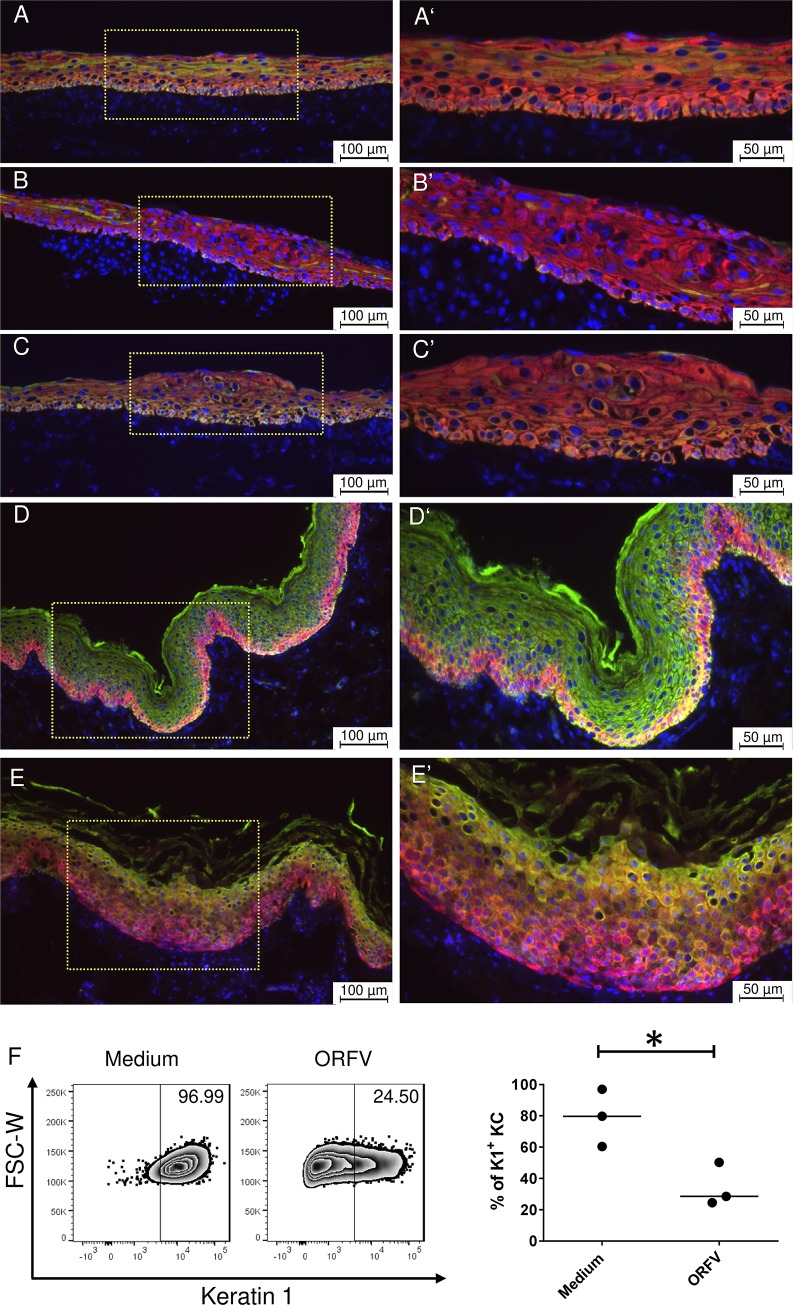
Reduced presence of K1 in ORFV-infected OTC, foreskin and KC culture. Representative fluorescence images showing the localization of K1 (green) and K14 (red) in OTC and foreskin (4 μm cryosections). K1 is localized in the suprabasal layer of non-infected OTC. The K14 signal is distributed all over the OTC epidermis (A and A’). In the long-term infected (d0-10) OTC the K1 signal is marginal, whereas the presence of K14 remains unchanged (B and B’). In the short-term infected OTC (d8-10) the K1 signal is also strongly reduced (C and C’). This K1 reduction seems to affect also the non-lesional area. In non-infected foreskin tissue a strong K1 signal is present in the suprabasal layer, whereas K14 staining is restricted to the *stratum basale* (D and D’). In the infected, CPE-affected sections, the fluorescence signal for K1 is distinctly reduced, while K14 is also visible in the suprabasal layer (E and E’). Each staining was done with OTC and foreskin from three different donors. Nuclei were stained with Hoechst 33342 (blue). Flow cytometry analysis shows a strong K1 reduction in ORFV-infected 2D-cultured KC (F). One representative flow cytometry plot and the statistical analysis of KCs derived from the same donor of three independent experiments is shown, comparing medium control to ORFV-infected samples, after testing for normal distribution using the one sample t-test.

As shown above, ORFV-induced cellular alterations were observed independently of the keratinocyte differentiation state in the OTC. Nevertheless, ORFV may interfere with keratinocyte differentiation. Thus, ORFV-infected KC were analyzed for the presence of K1 and K14 by immunofluorescence at the protein level and their transcription at the mRNA level (including K10 and loricrin). As observed before in non-infected OTC (Fig 1C), K1 was localized in the suprabasal layers, whereas K14 was present in basal and most suprabasal keratinocytes (Fig 5A and 5A’), in agreement with its long half-life [41]. When OTC were infected at day 0 for 10 days, the K14 fluorescence remained unaltered, whereas the K1 fluorescence was strongly reduced (Fig 5B and 5B’). Similar results were obtained when OTC were infected at day 8 for 2 days. Again, at day 10 the presence of K14 remained unchanged but K1 protein was reduced. The reduction of K1 extended even into the non-lesional area. (Fig 5C and 5C’). As seen before, in non-infected foreskin tissue cultured for 2 days in medium, K14 was localized to the *stratum basale*, whereas strong K1 staining was visible in the suprabasal layers of the epidermis (Fig 5D and 5D’). Upon ORFV infection, K1 level was remarkably diminished, whereas the distribution of K14 was no longer restricted to the basal layer (Fig 5E and 5E’).

To further confirm the K1 reduction caused by ORFV infection, we investigated the K1 presence of in 2D-cultured primary human foreskin keratinocytes by flow cytometry. Compared to non-infected KC, ORFV infection of KC cultures resulted in a significant decrease of K1-positive cells (Fig 5F). Taken together these data demonstrate that ORFV infection leads to a reduction of K1 in KC.

**Fig 6:**
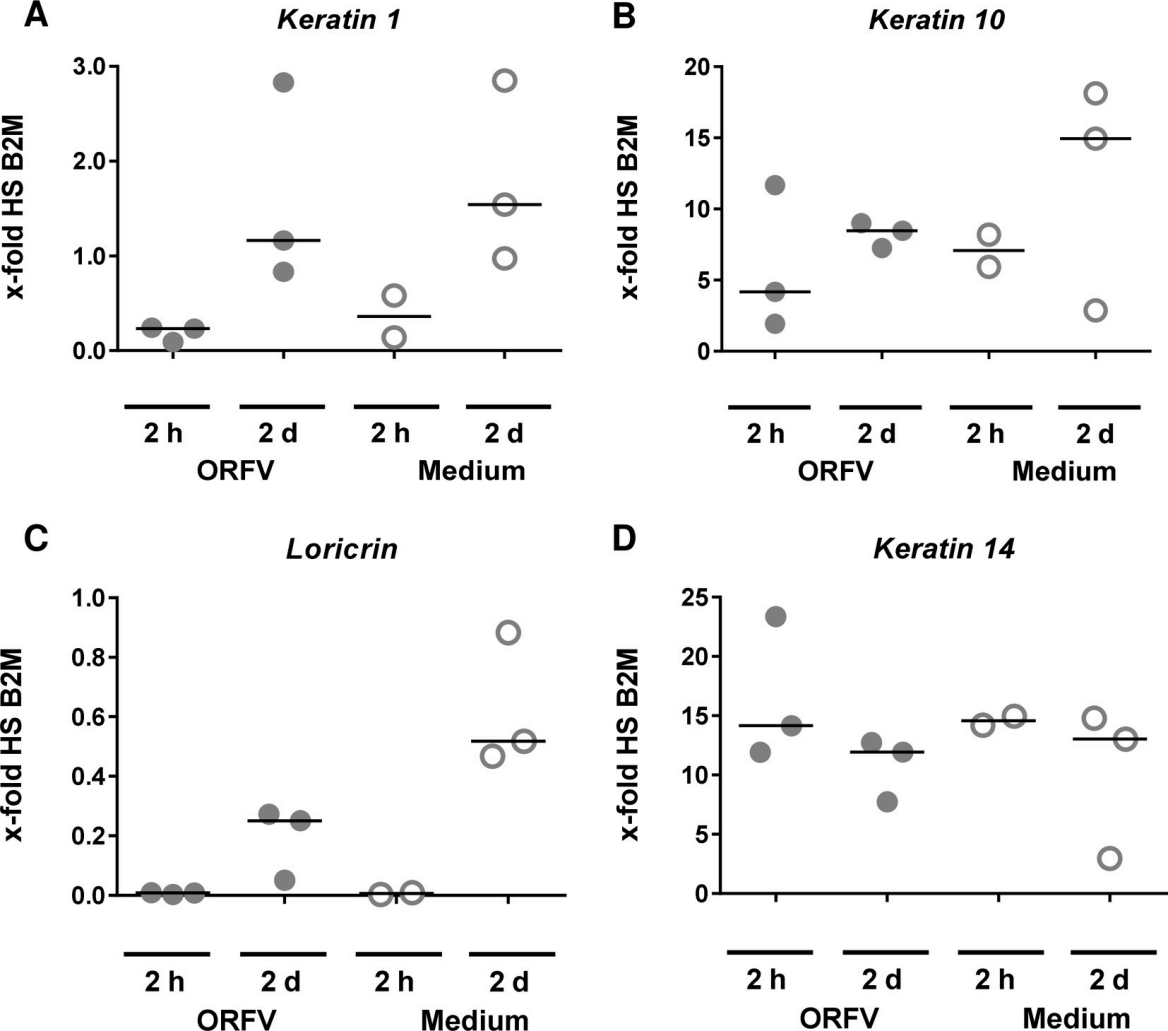
Down-regulation of *K1/K10* and *loricrin* in ORFV-infected OTC. Reduction of *K1*, *K10* and *loricrin* transcription is demonstrated by means of RT-qPCR and compared with non-infected tissue (A-C). Most importantly, *K14* transcription was unaltered in non-infected as well as in infected OTC (D). Transcription was analyzed 2 h and 48 h post infection (d8-10 infection) and calculated relative to human reference gene *beta-2-microglobulin (B2M)*. Each dot represents a separate OTC. Considering the fact that, in contrast to ORFV-specific genes, the differentiation markers *K1*, *K10*, and *loricrin* as well as *K14* are expressed both in ORFV-infected KC and in non-infected KC (which comprises the vast majority of the OTC), it is not appropriate to apply statistical analysis of expression strength.

To further evaluate whether keratin and *loricrin* transcription is down-regulated by ORFV infection, *K1*, *K10*, *K14*, and *loricrin* transcription was analyzed by RT-qPCR. Due to rapid cell death in 2D KC cultures caused by ORFV infection (MOI 3) Ct values are shown instead of relative comparison to reference gene expression (S6 Fig). In two out of three samples, the mRNA transcription of *K1* was reduced in ORFV-infected OTC (d8-10 infection) (Fig 6A). In addition, ORFV infection caused a decrease in *K10* expression (Fig 6B), that could also be seen when 2D KC cultures were infected with ORFV (S6 Fig). To exclude the possibility that CPE caused a general downregulation or silencing of keratins including *K14*, the relative expression of *K14* was determined and found to be unaffected by ORFV infection of OTC (Fig 6D) or of 2D KC cultures (S6 Fig), confirming the observation of unaltered K14 levels in ORFV-infected OTC described above (Fig 5). To analyze for a more general inhibition of terminal differentiation, we measured expression of the late differentiation marker *loricrin* [42,43]. *Loricrin* mRNA was down-regulated in ORFV-infected OTC (Fig 6C). In infected 2D KC cultures, however, *loricrin* transcription was low and not affected by ORFV infection (S6 Fig). At the same time, the observed differences between 2D and 3D culture support our notion that the 3D OTC model is the more appropriate experimental model to investigate ORFV infection.

Besides analysis of differentiation status we wished to characterize potential KC proliferation as well. To this end we analyzed the expression of *Ki-67* and *proliferating cell nuclear antigen* (*PCNA*) expressed by proliferating cells [38,44]. ORFV did neither affect the expression of *Ki-67* or *PCNA* in OTC (S5 Fig) nor in 2D KC cultures (S6 Fig), indicating that ORFV does not enhance KC proliferation. Thus, our data show for the first time that KC infection with ORFV inhibits *K1/K10* and *loricrin* expression on a transcriptional level in OTC.

## Discussion

We established an OTC model for human skin useful for studying the infection with ORFV, a zoonotic poxvirus whose pathogenesis in human skin is poorly characterized. Although numerous descriptions exist about histology of ORFV lesions [10,45], it is widely unknown how the infection establishes, where the virus can be detected and what effects are exerted on human skin cells, e.g. keratinocytes. Similar to cowpox virus infection in rat OTC [46], we used human skin OTC to monitor ORFV infection right at the beginning of OTC set up (d0-10) and near the termination of OTC (d8-10). In general, the histology of ORFV-specific CPE found in KC in lesions of human and animal origin was reproduced in OTC and was comparable to CPE in normal human skin such as foreskin. In distinct areas of CPE, ballooning degeneration of keratinocytes, vacuolization in the suprabasal layers and typical intra-plasmatic eosinophilic inclusions bodies were found [47–49], similar to cytological and histological patterns of human papilloma virus-induced lesions [50]. Ballooning in context with poxvirus infections is characterized by cell swelling and rounding of the cytoplasm and is caused by the rearrangement of the intermediate filaments of the cytoskeleton, in addition to further mechanisms [51,52].

Vacuolated keratinocytes that are present in ORFV infected OTC and foreskin (Fig 2C’ and 2E’) are reminiscent of koilocytes that are indicative and specifically related to human papilloma virus (HPV) infection [53]. Koilocytes are also found in bovine papilloma virus infection [54]. The appearance of koilocytes has so far not been described as a feature of ORFV induced CPE. However, the HPV preference for infection of immature basal cells fits well to the ORFV infection of proliferating cells and the appearance of tumor-resembling lesions. To define ORFV-specific induction and regular occurrence of koilocytes further research is needed.

In addition, at the restricted CPE sites in OTC, a thickening of the *stratum corneum* was detected at ORFV-infected areas, as described in sheep experimental skin infection [19]. ORFV induces proliferative skin lesions characterized amongst others by epidermal hyperplasia. Injection of vascular endothelial growth factor (VEGF)-like protein, a viral homolog of cellular VEGF, into normal skin increases the number of endothelial cells, leads to epidermal thickening and enhances keratinocyte numbers [15]. Interestingly, only at distinct areas of histologically overt CPE viral envelope protein was detectable in very few cells after the 10-day incubation period indicating an arrested virus presence. Obviously, only few virus-infected cells became integrated in the KC layer during OTC development, indicating that the majority of infected cells failed to participate in the OTC generation. In infected keratinocytes that participated, however, the virus persisted and permitted formation of an OTC. Consequently, more virus-positive KC were seen in CPE areas of short-term, near termination infected OTC (d8-10). Surprisingly, even replication-competent virus was present in the completed OTC infected directly at the onset as demonstrated by re-isolation of virus from OTC and foreskin in highly permissive cells. Thus, in some KC longevity of the input virus may be assumed, as no spread of infection was observed over wider areas of KC in OTC as well as in foreskin. The transcription of not only early and intermediate genes but also of a late gene (*B2L*) is evidence for at least one complete viral replication cycle. However, when considering the paucity of ORFV presence in infected OTC, one must conclude that even though ORFV is able to complete its replication cycle, it is unable to establish a productive spreading of the infection under these conditions. A similar situation was observed for Vaccinia virus in non-permissive cells resulting in expression of early and even late genes without final maturation and release of progeny virus [55,56]. The limitation of ORFV-infected KC in OTC reflects the strict localization of benign ORFV lesions in patients and the resolution of such lesions within a few weeks without scar formation [57,58]. The detection of parapoxvirus in normal skin or mucous membranes of healthy animals confirms the ability of the virus to persist without macroscopically visible tissue destruction or inflammation [59,60]. After scarification and infection of ovine skin, ORFV-specific protein was not found before 72 hours post infection [61]. This finding supports the failure of ORFV protein detection in incised human foreskin 48 hours after infection despite the presence of typical histological alterations and successful virus re-isolation.

Parapoxviruses are adapted to skin infection and perfectly manage to escape local immune responses [5]. Despite the preference of infecting proliferating cells, direct consequences of ORFV infection for human KC were unknown so far. Keratins form the cytoskeletal system consisting of intermediate filaments (IF) where K1 and K10 form the major IF cytoskeleton of postmitotic keratinocytes in the epidermis [41,62]. *K14* is expressed in the proliferating cells of the basal layer of stratified epithelia. In Molluscum Contagiosum Virus (MCV)-induced lesions, disruption of keratinization is reported with a preponderance of K14 and aberrant presence of K16 [63]. We could observe a strong reduction of K1/K10 presence in KC at ORFV-induced CPE sites in OTC and foreskin tissue, and expansion of K14 presence to suprabasal layers in infected foreskin explants, that, due to a suprabasal persistence of K14 as described for OTC also by others [39], did not become apparent in infected OTC. In addition, at the transcriptional level, a reduction of K1/K10 was detectable and K1 reduction by ORFV infection could be confirmed by flow cytometry with cultured KC. Interestingly inhibition of K1/K10 production was observed beyond the lesion-associated area, suggesting the involvement of a soluble mediator. A decrease of *K1*, *K10*, and *loricrin* expression was also observed in normal human keratinocytes and reconstructed human epidermis exposed to *Staphylococcus (S*.*) aureus* extracts [64]. In this study, the down-regulation of *K10* but not of *K1* induced by *S*. *aureus* extracts was dependent on IL-6. Addition of exogenous IL-6 to normal human keratinocytes was shown to down-regulate expression of *K10*, *K1*, and *loricrin* without affecting proliferation [64,65]. ORFV is able to induce IL-6 production [66–69]. Thus, it seems conceivable that ORFV induces IL-6 production that leads to the observed down-regulation of *K1*, *K10*, and *loricrin* in KC without having an impact on proliferation. In the pathogenesis of psoriasis, a decrease of K1 and K10 mRNA and protein level is induced by VEGF and associated with overexpression of the receptor VEGFR2 [70]. We found increasing expression of *VEGF-like protein* in ORFV-infected OTC (Fig 4A). It is tempting to speculate that ORFV-released VEGF-E [71], that exclusively binds to VEGFR2 [72], may also disturb *K1/K10* expression. Disruption or mutations of the keratin encoding genes can lead to loss of barrier function, reduced adhesion and hyperkeratotic disarrangement [32,73,74]. Whether the downregulation of *K1* and *K10* is involved in the ORFV-induced skin alterations observed in patients merits further investigations.

In summary, OTC offers the possibility to follow infection with a skin-adapted virus and to reveal virus-specific pathogenicity in human keratinocytes. Our study reveals the down-regulation of *K1/K10* and *loricrin* as hitherto unknown feature of epidermal ORFV infection.

## Supporting information

S1 Fig Schematic delineation of OTC preparation. A collagen mixture with fibroblasts was incubated for four days in culture medium. Subsequently, dispersed keratinocytes were added onto the collagen mixture. When the cells formed a monolayer, the plug was lifted on a metal grid to start differentiation at air-liquid interface conditions. The plug was incubated for 10 days.
(TIF)

S2 Fig Protocol of ORFV infection in OTC. Feeder cells mixed with collagen were incubated for four days before adding the keratinocytes. After two days incubation, either the infection was performed with ORFV at MOI 3 at a) day 0 or b) day 8. In total OTC was incubated for 10 days.
(TIF)

S3 Fig Analysis of OTC and skin structure by loricrin and K14 single/double staining. Representative fluorescence images showing the localization of loricrin (green) and K14 (red) in OTC and foreskin. Loricrin is localized in terminally differentiated keratinocytes in OTC (A) and foreskin (B). K14 is distributed all over the epidermis in OTC (C) and predominantly located to the cells of the *stratum basale* in foreskin (D). Loricrin and K14 double staining is shown in images E and F. Nuclei were stained with Hoechst 33342 (blue).
(TIF)

S4 Fig Detection of plaques following inoculation of ORFV–infected OTC and foreskin. Representative fluorescence images show virus-specific plaques in permissive KOP cells (A and B). ORFV envelope specific staining is shown in green. The nuclei were stained with Hoechst 33342 (blue).
(TIF)

S5 Fig *Ki-67* and *PCNA* expression in ORFV-infected OTC. Expression of *Ki-67* and *PCNA* is shown by means of RT-qPCR and compared with non-infected tissue (A-B). Transcription was analyzed 2 h and 48 h post infection (d8-10 infection) and calculated relative to human reference gene *beta-2-microglobulin (B2M)*. Each dot represents a separate OTC. For statistical analysis the normal distribution was calculated with the Shapiro-Wilk test. The data derived from 2-d ORFV infection were normally distributed, they were tested for statistical significance in comparison to the reference (data: medium 2 d) with the one sample t test.
(TIF)

S6 Fig Down-regulation of *K10* in ORFV-infected 2D keratinocyte culture. Expression of *K1*, *K10*, *loricrin*, *K14*, *Ki-67* and *PCNA* transcription was analysed by means of RT-qPCR and compared with non-infected tissue (A-F). Transcription was analyzed 2 h and 48 h post infection. Threshold cycle (Ct) values are shown since relative quantification was hampered by rapid cell death in 2D KC cultures caused by ORFV infection at the MOI of 3 leading to low expression of reference gene B2M. Ct values were determined using cDNA corresponding to 0.5 ng (K14, PCNA), 5 ng (K1, K10, Ki-67) or 50 ng (loricrin) reverse-transcribed RNA. Cells from three different donors were tested. For statistical analysis the normal distribution was calculated with the Shapiro-Wilk test. The ORFV data (2 d) were normally distributed, they were tested for statistical significance in comparison to the reference (data: medium 2 d) with the one sample t test. For K10 a significant (*, p<0.05) reduction can be seen.
(TIF)
