# Supplementary figures and images for: Orf virus (ORFV) infection in a three-dimensional human skin model: Characteristic cellular alterations and interference with keratinocyte differentiation
Source: PLoS One. 2019 Jan 30;14(1):e0210504. doi: 10.1371/journal.pone.0210504 (PMC6353139; doi:10.1371/journal.pone.0210504)

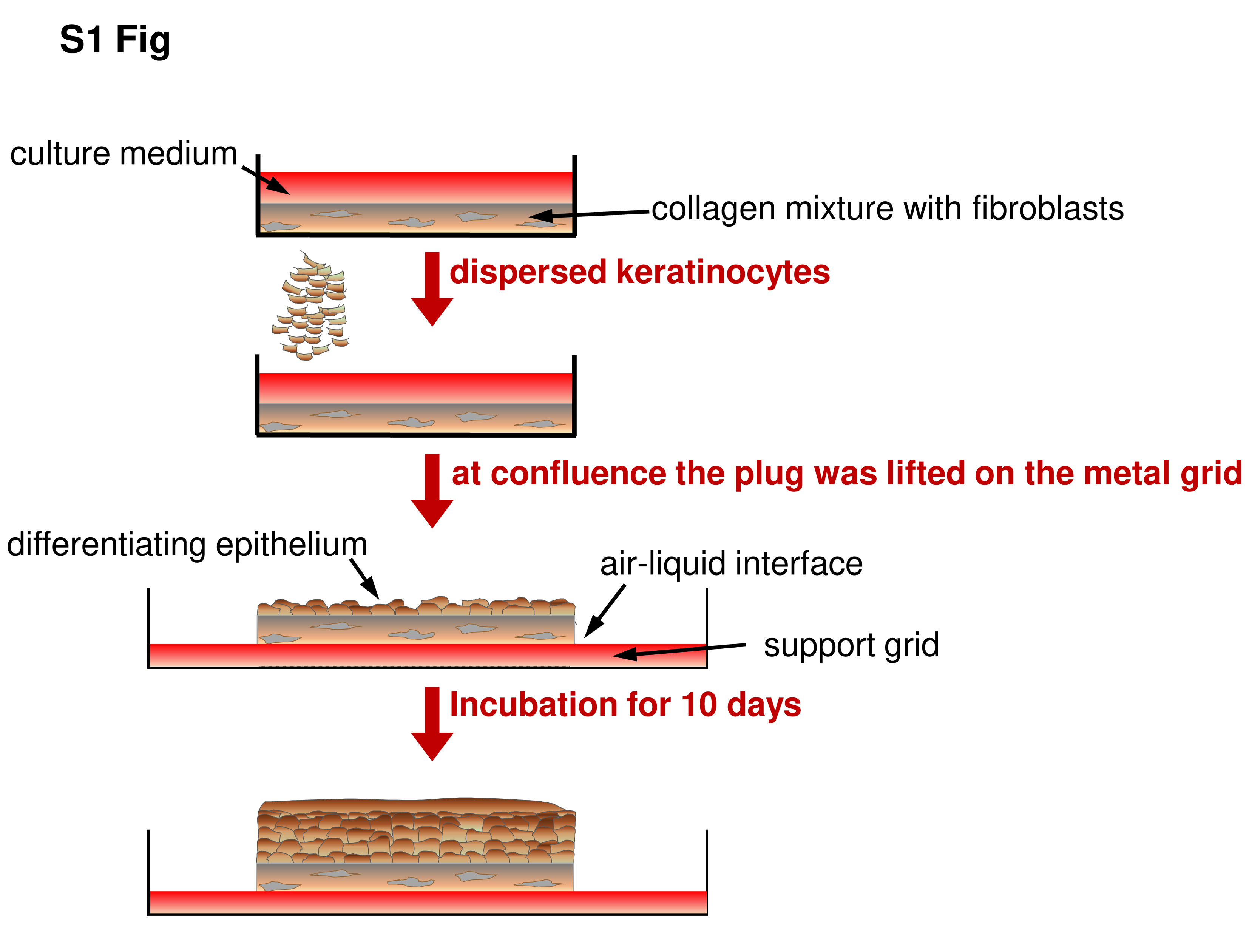

Supplement: S1 Fig — A collagen mixture with fibroblasts was incubated for four days in culture medium. Subsequently, dispersed keratinocytes were added onto the collagen mixture. When the cells formed a monolayer, the plug was lifted on a metal grid to start differentiation at air-liquid interface conditions. The plug was incubated for 10 days. (TIF) [file pone.0210504.s001.tif]

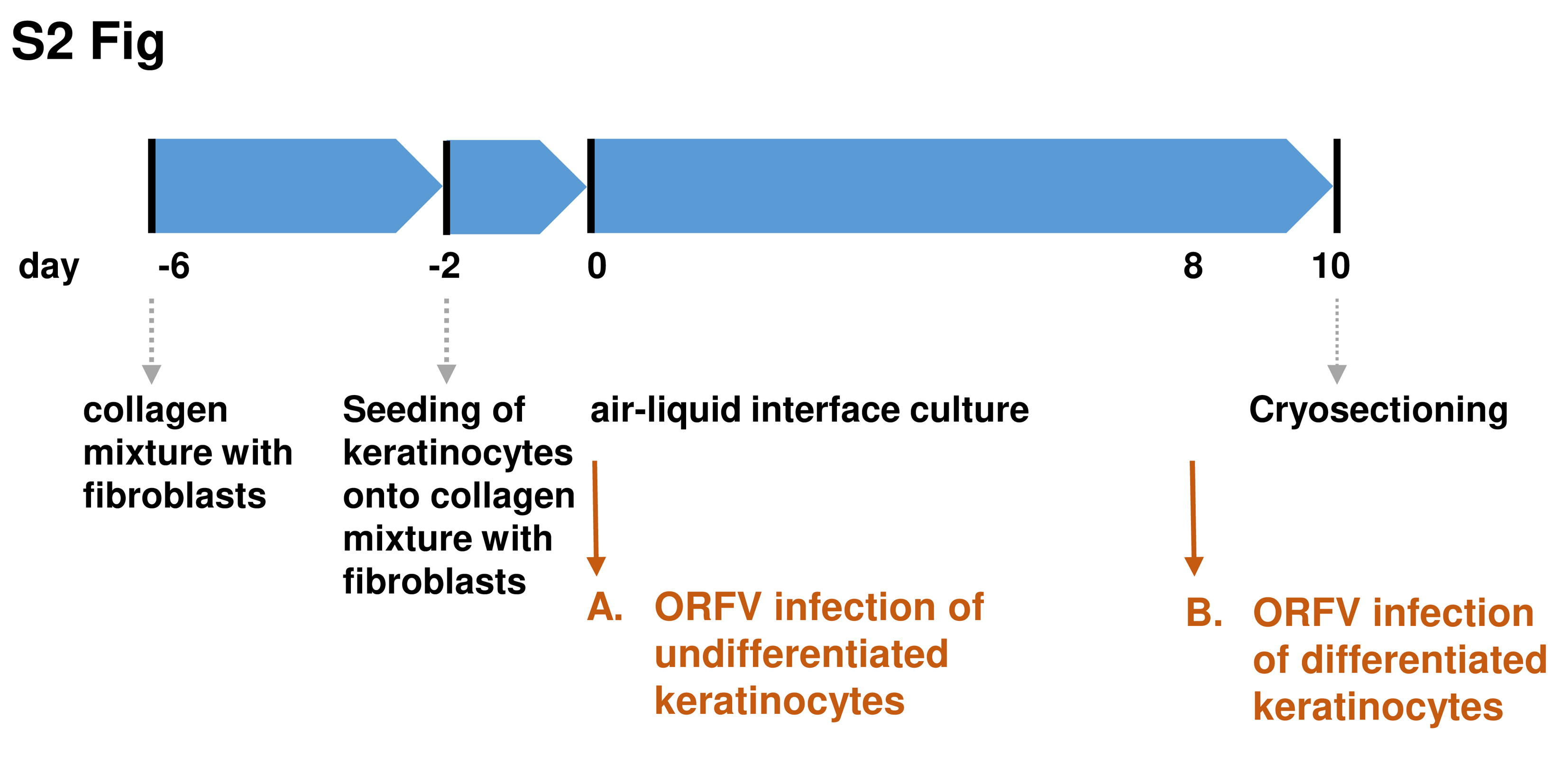

Supplement: S2 Fig — Feeder cells mixed with collagen were incubated for four days before adding the keratinocytes. After two days incubation, either the infection was performed with ORFV at MOI 3 at a) day 0 or b) day 8. In total OTC was incubated for 10 days. (TIF) [file pone.0210504.s002.tif]

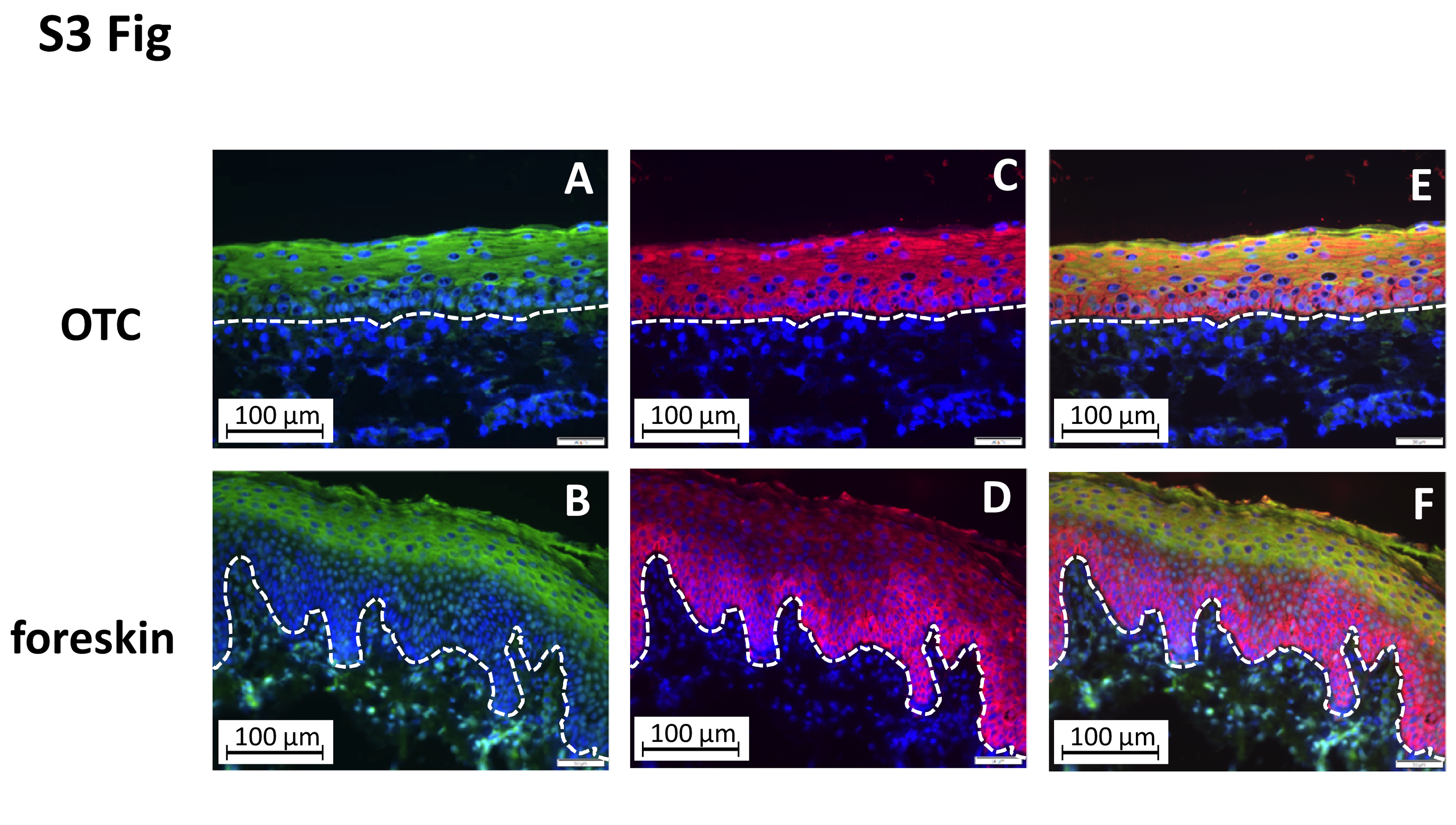

Supplement: S3 Fig — Representative fluorescence images showing the localization of loricrin (green) and K14 (red) in OTC and foreskin. Loricrin is localized in terminally differentiated keratinocytes in OTC (A) and foreskin (B). K14 is distributed all over the epidermis in OTC (C) and predominantly located to the cells of the stratum basale in foreskin (D). Loricrin and K14 double staining is shown in images E and F. Nuclei were stained with Hoechst 33342 (blue). (TIF) [file pone.0210504.s003.tif]

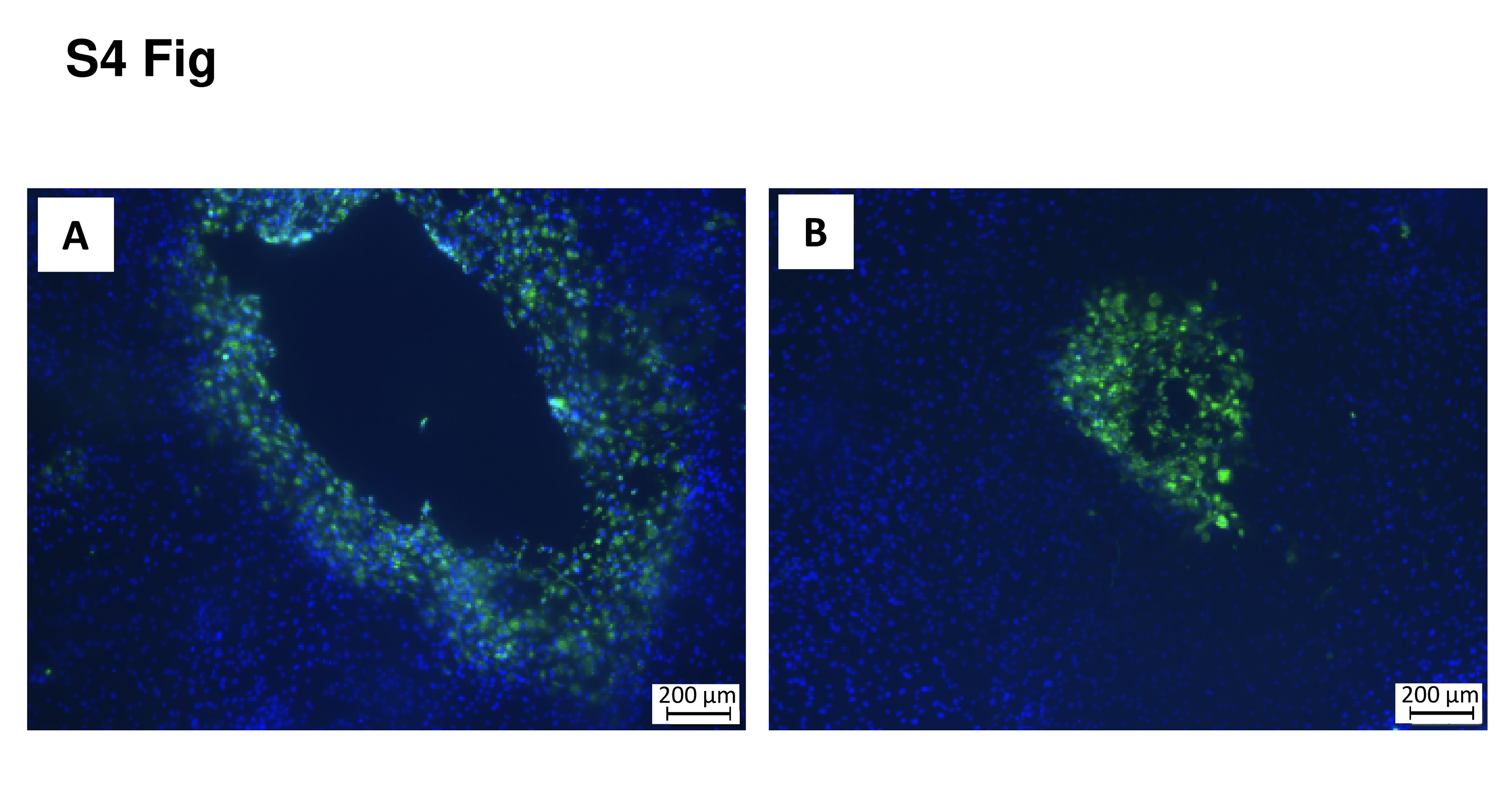

Supplement: S4 Fig — Representative fluorescence images show virus-specific plaques in permissive KOP cells (A and B). ORFV envelope specific staining is shown in green. The nuclei were stained with Hoechst 33342 (blue). (TIF) [file pone.0210504.s004.tif]

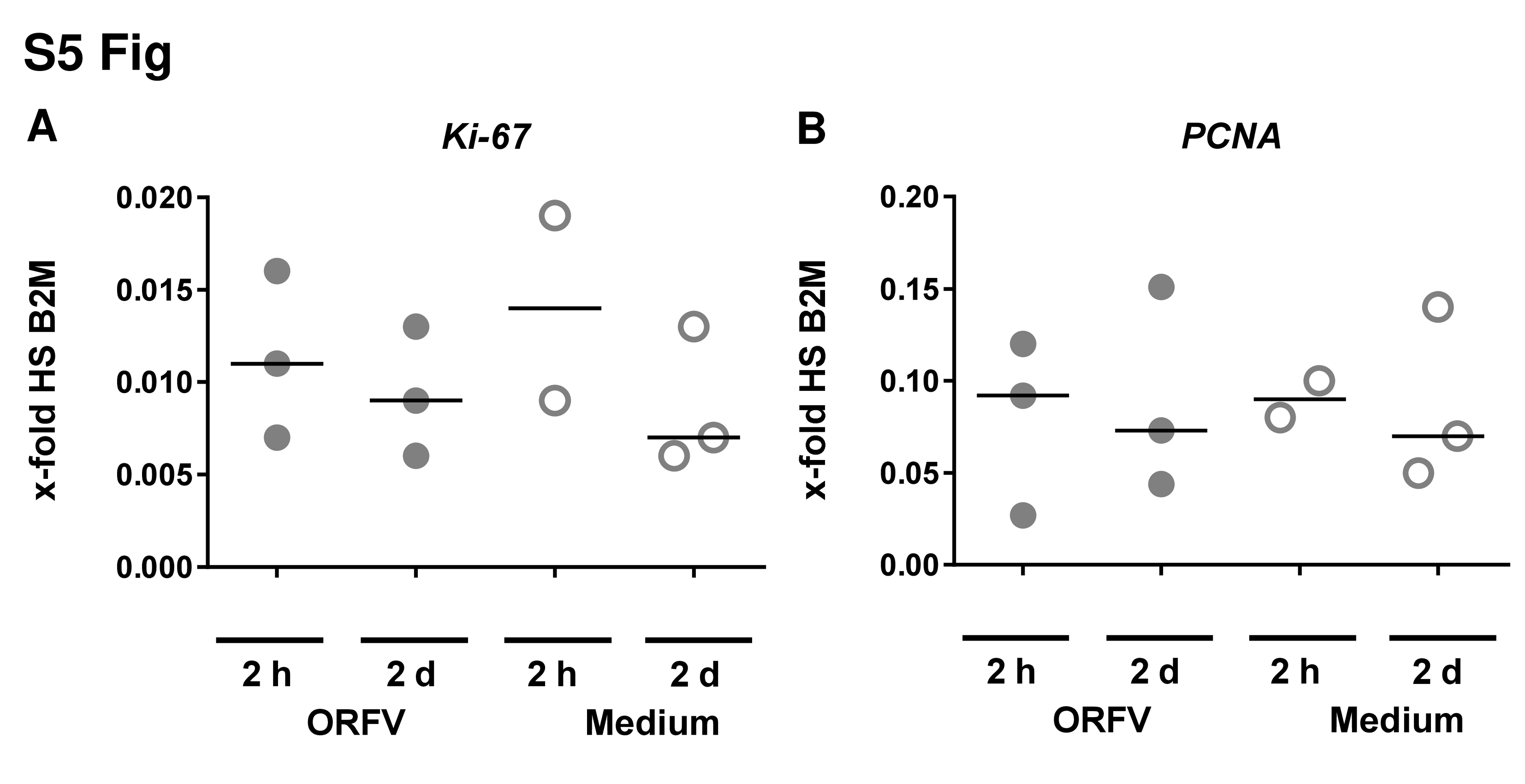

Supplement: S5 Fig — Expression of Ki-67 and PCNA is shown by means of RT-qPCR and compared with non-infected tissue (A-B). Transcription was analyzed 2 h and 48 h post infection (d8-10 infection) and calculated relative to human reference gene beta-2-microglobulin (B2M). Each dot represents a separate OTC. For statistical analysis the normal distribution was calculated with the Shapiro-Wilk test. The data derived from 2-d ORFV infection were normally distributed, they were tested for statistical significance in comparison to the reference (data: medium 2 d) with the one sample t test. (TIF) [file pone.0210504.s005.tif]

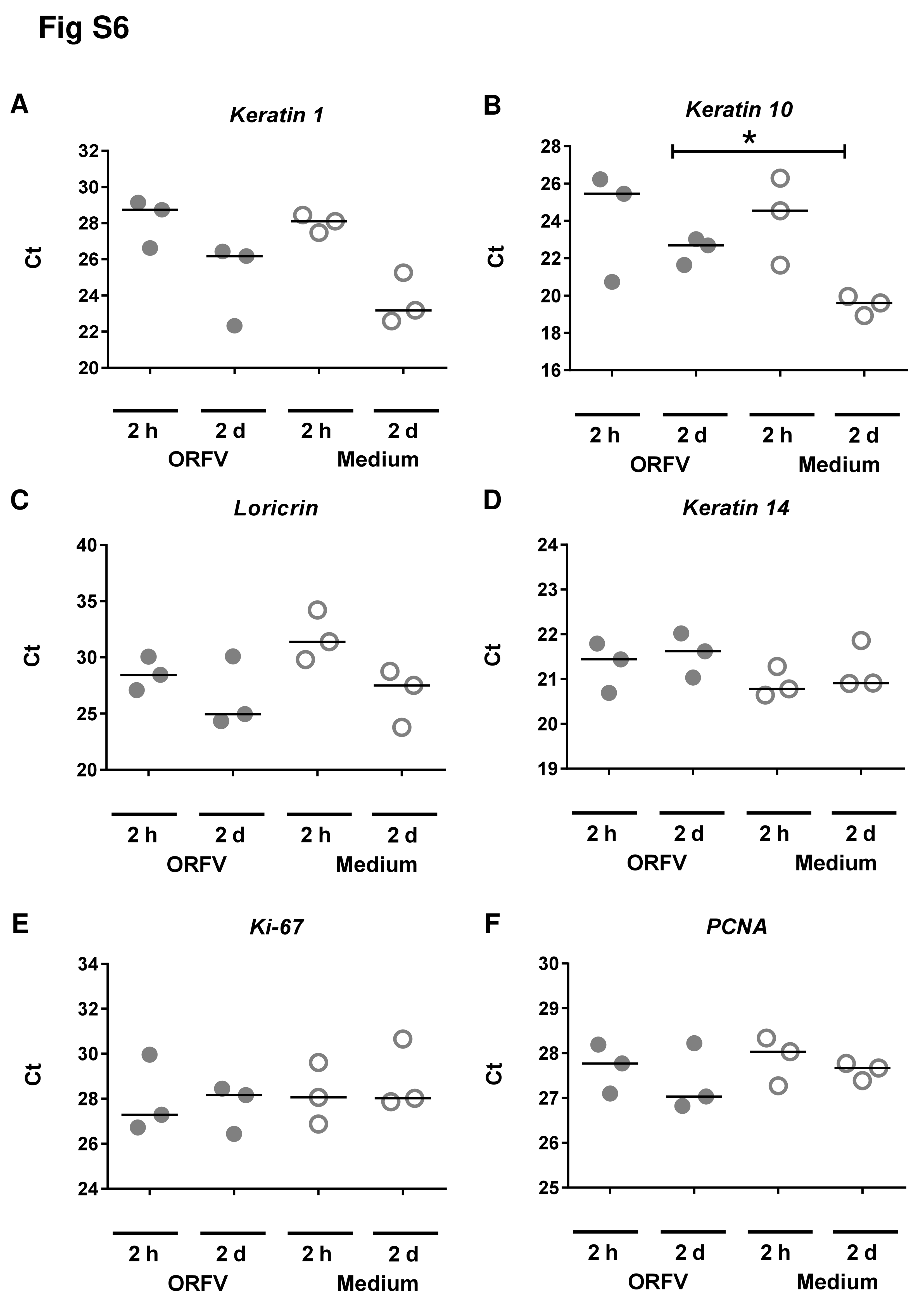

Supplement: S6 Fig — Expression of K1, K10, loricrin, K14, Ki-67 and PCNA transcription was analysed by means of RT-qPCR and compared with non-infected tissue (A-F). Transcription was analyzed 2 h and 48 h post infection. Threshold cycle (Ct) values are shown since relative quantification was hampered by rapid cell death in 2D KC cultures caused by ORFV infection at the MOI of 3 leading to low expression of reference gene B2M. Ct values were determined using cDNA corresponding to 0.5 ng (K14, PCNA), 5 ng (K1, K10, Ki-67) or 50 ng (loricrin) reverse-transcribed RNA. Cells from three different donors were tested. For statistical analysis the normal distribution was calculated with the Shapiro-Wilk test. The ORFV data (2 d) were normally distributed, they were tested for statistical significance in comparison to the reference (data: medium 2 d) with the one sample t test. For K10 a significant (*, p<0.05) reduction can be seen. (TIF) [file pone.0210504.s006.tif]
